# Effects of DEHP, DEHT and DINP Alone or in a Mixture on Cell Viability and Mitochondrial Metabolism of Endothelial Cells In Vitro

**DOI:** 10.3390/toxics10070373

**Published:** 2022-07-04

**Authors:** Kelly Poitou, Tiphaine Rogez-Florent, Anaïs Dirninger, Cécile Corbière, Christelle Monteil

**Affiliations:** Université de Rouen Normandie UNIROUEN, UNICAEN, ABTE, 76000 Rouen, France; kelly.poitou1@univ-rouen.fr (K.P.); anais.dirninger@etu.univ-rouen.fr (A.D.); cecile.corbiere@univ-rouen.fr (C.C.)

**Keywords:** DEHP, DEHT, DINP, mixture effects, human microvascular endothelial cells, in vitro, mitochondrial dysfunction

## Abstract

Plasticizers are chemicals in high demand, used in a wide range of commercial products. Human are exposed through multiple pathways, from numerous sources, to multiple plasticizers. This is a matter of concern, as it may contribute to adverse health effects. The vascular system carries plasticizers throughout the body and therefore can interact with the endothelium. The aim of the study was to evaluate the in vitro toxicity on endothelial cells by considering the individual and the mixture effects of bis-(2-ethylhexyl) phthalate (DEHP), diisononyl phthalate (DINP) or bis-(2-ethylhexyl) terephthalate (DEHT). In this study, their cytotoxicity on HMEC-1 cells was evaluated on cell function (viability, cell counting, total glutathione and intracellular adenosines) and mitochondrial function (mitochondrial respiration). Results showed cellular physiological perturbations induced with all the condition tested, excepted for DEHT. Plasticizers induced a cytotoxicity by targeting mitochondrial respiration, depleting mitochondrial ATP production and increasing glycolytic metabolism. Additionally, delayed effects were observed between the cellular and the mitochondrial parameters. These results suggest that endothelial cells could go through a metabolic adaptation to face plasticizer-induced cellular stress, to effectively maintain their cellular processes. This study provides additional information on the adverse effects of plasticizers on endothelial cells.

## 1. Introduction

With global consumption reaching 7.5 million tons annually, plasticizers are chemicals in high demand [1]. Plasticizers are chemical additives of low molecular weight. These compounds are used to soften and to increase the flexibility of plastic materials [2]. Their uses broaden over time to meet the growing industrial demand to the extent that plasticizers are an ubiquitous part of contemporary lifestyle. They have been reported in a wide range of commercial products, such as food packaging, toys, medical devices and protective gloves [3,4,5,6]. Although plasticizer consumption is tremendous, their use is not without risks.

Plasticizers can leach out during product use because they are not covalently bound. Thus, plasticizer leaching from products is at risk of exposure, although this is unintentional. Human exposure can occur through multiple pathways of exposure, including oral, respiratory, dermal and parenteral routes [7,8,9,10]. This is a matter of particular concern because human exposure to plasticizers has been related to adverse health effects. Many epidemiological and experimental studies have focused on evaluating their reproductive disorders [11,12,13,14,15,16,17,18,19,20]. Lately, studies have reported the association between exposure to plasticizers and cardiovascular risk factors (e.g., high blood pressure, arrythmias, atherosclerosis) [21]. More recently, cardiovascular mortality was attributed to phthalate exposure [22]. However, investigation on the toxicological effects of plasticizers on the cardiovascular system remain limited, particularly considering that various medical devices containing a high plasticizer content can expose the circulatory system [9,23,24].

Among the few studies conducted on the circulatory system, bis-(2-ethylhexyl) phthalate (DEHP) is by far the most studied plasticizer, even though there is a diversity of plasticizers on the market. DEHP was shown to induce vascular injury in orally exposed C57/BL6 mice through alterations in vascular tone [25]. Moreover, DEHP contributed to inflammation by increasing the serum level of pro-inflammatory cytokines in ApoE^−^/^−^ mice and in human umbilical vein endothelial cells [26,27]. Vascular damage induced by plasticizers could also be mediated by oxidative stress on endothelial cells, as seen with the increasing level of reactive oxygen species (ROS) in EA.hy926 cells exposed to mono-2-ethylhexyl phthalate (MEHP), the main metabolite of DEHP [28,29]. Inflammation and oxidative stress are two mechanisms that are plausible contributors of atherosclerosis. DEHP-promoted atherosclerosis was reported in intraperitoneally exposed ApoE^−^/^−^ mice through LDL oxidation within the arterial wall and macrophage infiltrations along with the disruption in lipid metabolism [26]. Altogether, the above-mentioned results lead to think that exposure to plasticizers could mediate endothelial cell dysfunction. Indeed, DEHP exposure modified the expression of adhesion molecules in ApoE^−^/^−^ mice and in human umbilical vein endothelial cells [26,27]. Besides, endothelial cell injury was observed in vitro following DEHP exposure with an imbalance in the antioxidant status [28,30]. Endothelial dysfunction could be related to mitochondrial alteration, as it is a well-known target of oxidative stress [31]. The effects of plasticizers on the mitochondria were mostly studied considering the mechanism of cell death with mitochondrial outer membrane permeabilization [28,30,32]. However, the mitochondrial energetic status following a cellular stress induced by plasticizers remain limited despite its crucial role for cell functions.

Furthermore, most toxicological studies are not representative of real-life exposure. On the one hand, plasticizers have mostly been evaluated individually although human biomonitoring studies have emphasized that populations are exposed to a mixture of plasticizers [33,34]. Hence, the effects of individual plasticizers is possibly modified when in mixture because chemicals can interact with each other as revealed with the cumulative effect of a mixture of five anti-androgenic phthalates on the reproductive tract development in male rats [35]. Yet, the exposure to multiple plasticizers is rarely taken into account apart from the reproductive system [36,37,38]. On the other hand, the REACH legislation framework has initiated the replacement of classified phthalates (DEHP, benzyl butyl phthalate, diisobutyl phthalate, dibutyl phthalate, dipropyl phthalate and dicyclohexyl phthalate) by alternative plasticizers, such as long-chain phthalates (e.g., diisononyl phthalate (DINP)) and non-phthalate plasticizers (e.g., bis-(2-ethylhexyl) terephthalate (DEHT)) [34,39]. However, the substitution of plasticizers does not necessarily ensure absence of toxicity. For example, DINP promoted hypertension in C57/BL6 mice following oral exposure by contributing to vascular endothelial dysfunction through a decrease in both endothelial nitric oxide synthase expression and nitric oxide production [40].

Experimental data on the effects of plasticizer mixture on the vascular system are scarce even though it is a known target of the plasticizers. To our knowledge, only a recent in vivo study was conducted to investigate the effects of plasticizer mixture on the vascular system [41]. However, it investigated the blood–brain barrier, not the peripheral vasculature. Thus, it is necessary to fill the remaining gaps because all the above-mentioned detrimental effects of plasticizers on the vascular system encourage us to think that direct exposure to plasticizers could result in endothelial dysfunction through an oxidative mechanism or a mitochondrial impairment. To overcome this knowledge gap, this work aimed to assess in vitro the toxicological effects of three representative plasticizers, alone and in a mixture, on a human microvascular endothelial cell line (HMEC-1). The three plasticizers were selected based on a previous work performed from gloves [6] and included DEHP a well-known reproductive toxicant, along with two alternative plasticizers, DINP and DEHT, as they constitute a representative group of the most used plasticizers while also including traditional and alternative plasticizers [42,43,44]. We first compared cell viability, total intracellular glutathione and adenosines levels following continuous (24, 48 and 72 h) or repeated (3 h per day for three consecutive days: J1, J2 and J3) exposure in order to investigate the recovery capacity of cells after phthalate exposure. Then, we determined whether phthalate exposure induces mitochondrial dysfunction. We discovered a strong and early effect of DEHP on mitochondrial function, with a shift between mitochondrial respiration and anaerobic glycolysis, while, overall, the ATP levels remained unaltered until a decrease in cell viability was seen, suggesting a metabolic adaptation of endothelial cells. DINP exposure induced a late effect and DEHT did not induce any alteration, whatever the exposure time. These results provide new insights into the cellular effects underlying phthalates endothelial toxicity.

## 2. Materials and Methods

### 2.1. Human Microvascular Endothelial Cell Line (HMEC-1) Culture

HMEC-1 cell line was provided by American Type Culture Collection (ATCC^®^, CRL-3243^TM^). These cells are human microvascular endothelial cells that were immortalized by transfection with simian virus 40. This cell line features a non-pathological phenotype with applications for in vitro toxicological studies [45,46]. HMEC-1 cells were cultured in MCDB 131 medium (Gibco, Fisher Scientific, Illkirch, France) supplemented with 10% fetal bovine serum (FBS, Gibco), 10 mM L-glutamine (Gibco), 10 ng/mL recombinant human epidermal growth factor (EGF, Gibco), 10 U/mL penicillin and 10 µg/mL streptomycin (Gibco) and with 1 µg/mL hydrocortisone, obtained from Sigma-Aldrich (Merck, Saint-Quentin-Fallavier, France). Cells were growth in culture flasks purchased from BD Falcon (Corning, NY, USA) and were incubated with 5% CO_2_ at 37 °C. At confluency, cells were rinsed with Dulbecco’s phosphate-buffered saline (D-PBS) and cell passage was operated using 0.05% trypsin-EDTA (Gibco). Cells were counted after a dilution in 0.1% trypan blue solution (Sigma-Aldrich). For the experiments, cells were seeded in either 96-well, 6-well microplates (BD Falcon) or 24-well microplates (Seahorse, Agilent Technologies, Santa-Clara, CA, USA).

### 2.2. Studied Plasticizers

**Figure 1:**
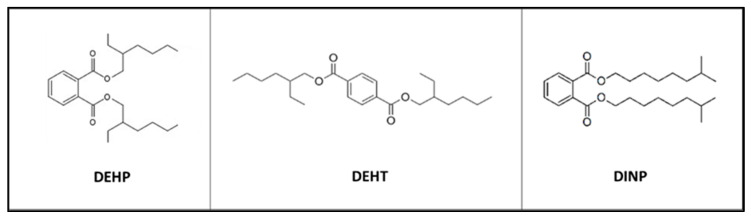
Topological structure of the studied plasticizers. Bis-(2-ethylhexyl) phthalate (DEHP), bis-(2-ethylhexyl) terephthalate (DEHT) and diisononyl phthalate (DINP).

The three plasticizers were all purchased from Sigma-Aldrich and included DEHP (CAS number 117-81-7), DEHT (CAS number 6422-86-2) and DINP (CAS number 28553-12-0) (Figure 1). DEHP is a well-recognized regulated plasticizer due to its reproductive toxicity (category 1 H360). DEHT and DINP are two alternative plasticizers to DEHP because they are considered of lower toxicity. These three plasticizers were selected based upon previous experiments carried out in our laboratory [6].

### 2.3. Solution Preparations

For each plasticizer, a stock solution was prepared by gravimetry at a concentration of 10 g/L in dimethyl sulfoxide (DMSO) obtained from Sigma-Aldrich. Intermediates solutions were prepared at a targeted concentration of 1500 mg/L in DMSO for both plasticizers individually and in mixture. All treatment solutions (DEHP, DEHT, DINP and the mixture) were prepared extemporaneously by diluting intermediate solutions in MCDB medium at a final concentration of 15 mg/L with 1% DMSO for each condition. For the experiments, 1% DMSO was used as the solvent control.

### 2.4. Reagents and Standards

All reagents used were provided by Sigma-Aldrich except the following chemicals: ethylenediaminetetraacetic acid (EDTA) was obtained from Prolabo (VWR, Fontenay-sous-Bois, France), β-Nicotinamide adenine dinucleotide 2′-phosphate reduced tetrasodium salt hydrate (NADPH) was provided by Roche (Mannheim, Germany), sodium potassium tartrate tetrahydrate was purchased from Carlo-Erba (Val-de-Reuil, France) and, hydrochloric acid and sodium hydroxide were obtained from VWR (Fontenay-sous-Bois, France). Bradford reagent was provided respectively by Bio-Rad (Hercules, CA, USA). Ultrapure water was supplied by a water purification system from ELGA LabWater (Veolia, Saint-Maurice, France).

### 2.5. Plasticizer Treatments and Exposure Procedures on HMEC-1 Cells

Cells were treated at a concentration of 15 mg/L for both plasticizers tested individually and in mixture (DEHP, DEHT and DINP). The tested concentration of 15 mg/L (equal to about 40 µM) was chosen regarding the adverse effects of plasticizers on the cardiovascular system described by previous toxicological studies [30,47]. The cytotoxicity of MEHP, the main metabolite of DEHP, was reported on endothelial cells in the same range of concentrations. Indeed, oxidative stress and mitochondrial damage were noted for tested concentrations between 12.5 and 100 µM [30]. Moreover, the implication of DEHP in mitochondrial dysfunction was reported, as a metabolic reprogramming in cardiomyocytes was described at a concentration of 50 mg/L [47]. Since DMSO and the culture medium differ by their polarity, their solubility was checked beforehand in order to obtain homogeneous solutions. Cell treatments were practically feasible at the chosen concentration because no precipitation occurred during the preparation of the treatment solutions. The studied plasticizers were tested on cells following two distinctive exposure procedures to evaluate respectively continuous and repeated exposures for the following toxicological endpoints: cell viability, total intracellular glutathione (total glutathione) content and cellular adenosine amounts. As for mitochondrial metabolism, it was determined following continuous exposure solely. For continuously exposure, cells were constantly exposed with each treatment solution during all the experiment, with exposure times ranging from 24 h to 72 h. For repeated exposure, cells were intermittently exposed with an exposure time of 3 h a day with each condition (from J1 to J3). Exposures were then stopped by removing the treatment solutions and by replacing it by fresh culture medium until the following exposure.

### 2.6. Cell Viability

For the evaluation of the cell viability, HMEC-1 cells were seeded in 96-well microplates at a density of 2.0 × 10^4^ cells/well (6 × 10^4^ cells/cm^2^) in 100 µL of culture medium. Cells were treated at confluency with 100 µL of each treatment solution after removing the culture medium in each well. Cell viability was assessed by spectrophotometry using a 3-(4,5-dimethylthiazol-2-yl)-2,5-diphenyltetrazolium bromide (MTT) assay. At the end of each exposure time, 10 µL of a MTT solution (5 mg/mL prepared in D-PBS) was added to each well. After three hours of incubation away from light, 100 µL of a sodium dodecyl sulfate solution (10% prepared in 0.01 N hydrochloric acid) were put in each well to dissolve reduced formazan crystals and microplates were then incubated overnight. The following day, absorbance measurements were performed at 570 nm using a microplate reader (SAFAS Xenius, SAFAS Monaco, Monaco). For each treatment group, the mean of at least five independent cell culture preparations was calculated. Cell viability is expressed as the relative percentage to the solvent control (DMSO 1%), which was standardized at 100%.

### 2.7. Cell Counting

For cell counting, HMEC-1 cells were seeded in 6-well microplates at a density of 4.5 × 10^5^ cells/well (5 × 10^4^ cells/cm^2^) in 2 mL of culture medium. At confluency, the culture medium was removed and cells were treated with 2 mL of each treatment solution. Cell counting was based on the trypan blue exclusion method following 24, 48 and 72 h of exposure. For each treatment group, cells were counted after a dilution in 0.1% trypan blue solution, using Malassez cells. Cell counting results are expressed in percentage of viable cells. The data are expressed as the mean ± SD of at least four independent cell culture preparations, for each different experimental condition.

### 2.8. Total Intracellular Glutathione Content

For the evaluation of total intracellular glutathione content, HMEC-1 cells were seeded in 6-well microplates at a density of 4.5 × 10^5^ cells/well (5 × 10^4^ cells/cm^2^) in 2 mL of culture medium. Then, the culture medium was removed either at confluency (for continuously exposures) or at sub-confluency (for repeated exposures) and cells were treated with 2 mL of each treatment solution. A two-step extraction with perchloric acid (PCA) obtained from Prolabo (VWR, Fontenay-sous-Bois, France) was conducted. Into each well, 500 µL (an aqueous PCA solution 1 N) were added and cells extracts were collected with a cell scraper. For each sample, the solution was collected in the microtube and was centrifugated at 4 °C (13,500× *g* for 5 min). Cell protein pellets were collected and resuspended in sodium hydroxide (an aqueous NaOH solution 1 N) for the protein assay. The supernatant was neutralized with an equal volume of carbonate of potassium (an aqueous K_2_CO_3_ solution 2 M) (Sigma-Aldrich) and was centrifugated at 4 °C (13,500× *g* for 5 min). The evaluation of total intracellular glutathione content in cells following plasticizer exposures was performed using the enzymatic recycling method with 5,5′-dithiobis(2-nitrobenzoic acid) (DTNB) reagent in a 96-well microplate. A volume of 10 µL of sample was added along with 30 µL of a phosphate buffer solution of pH 7.4 (143 mM NaH_2_PO_4_ and 6.31 mM EDTA prepared in ultrapure water) and 200 µL of reactive buffer prepared in a phosphate buffer solution (0.31 mg/mL DTNB and 0.23 mg/mL NADPH). A volume of 40 µL of 8.5 U/mL glutathione reductase was added briefly before evaluating enzymatic kinetic by measuring TNB formation at 405 nm during 2 min 40 s with a microplate reader (Tecan Spark^®^, Männedorf, Switzerland). For each treatment group, the content of total intracellular glutathione was normalized to the protein amount of the corresponding sample (Lowry protein assay) and it was standardized with the solvent control (DMSO 1%). The data are expressed as the mean ± SD of at least four independent cell culture preparations, for each different experimental condition.

### 2.9. Intracellular Adenosine Amounts

Intracellular adenosine amounts (ATP, ADP and AMP respectively) were measured on the supernatant of the cellular extract after PCA extraction, as described above. The quantification of cellular adenosines was made using a high-performance liquid chromatography coupled with diode array detector (HPLC-DAD) method previously developed and validated in our laboratory for the simultaneous determination of ATP, ADP and AMP in biological samples [48]. Analyses were performed using the 1260 Infinity II LC System (Agilent Technologies, Santa Clara, CA, USA). Chromatographic separation was achieved on a Poroshell 120 EC-C18 column (150 mm × 3 mm, 2.7 μm i.d.) with an EC-C18 guard (3 mm) both at 20 °C. One microliter of each sample was injected. Adenosines were separated in isocratic elution consisting of 50 mM potassium hydrogen phosphate of pH 6.80. The flow rate was kept at 0.6 mL/min. Detection of all three adenosines was performed at 254 nm. Data were processed using OpenLab CDS LC ChemStation software (Agilent). The concentrations of intracellular adenosines in samples were determined using calibration curve obtained with the standard solutions (0.2–10 µM respectively for ATP, ADP and AMP). Each adenosine concentration was normalized to the amount of protein of the corresponding sample and it was expressed in nmol/mg of protein. For each treatment group, the ATP/ADP ratio was determined and standardized with the solvent control (DMSO 1%). The data are expressed as the mean ± SD of at least four independent cell culture preparations, for each different experimental condition.

### 2.10. Cell Metabolism Assays

Oxygen consumption rate (OCR) and extracellular acidification rate (ECAR) were measured using a Seahorse XF24 extracellular flux analyzer (Agilent technologies), according to the manufacturer’s instructions. Cells were seeded in XF24 culture microplates for 96 h before the measurements in order to allow the cells to be exposed to the plasticizers as a function of time (3 h, 24 h or 48 h). The optimum number of cells/well was determined to be 25,000 cells/well. After the different exposures, the cells were then washed free of the plasticizers or solvent (DMSO 1%) and incubated with a phenol red-free DMEM-based medium supplemented with 10 mM Glucose, 1 mM pyruvate and 2 mM glutamine in a CO_2_-free incubator for 60 min before the Seahorse assay. A stress test was performed with addition of oligomycin (8 µg/mL) after the measurement of the baseline OCR (3 cycles, 24 min), to inhibit the ATP synthase and estimate basal respiration coupled to ATP synthesis (3 cycles, 24 min). To determine the maximal OCR, 1 µM carbonyl cyanide-4-(trifluoromethoxy)phenylhydrazone (FCCP) was injected (3 cycles, 24 min). Finally, a combination of antimycin A (AA, 2 µg/mL) and rotenone (ROT, 5 µM) was injected to inhibit electron flux (3 cycles, 24 min). Data analysis was conducted using Wave Software (Version 2.6.3.5; Agilent Technologies). The data are expressed as the mean ± SD of at least three independent cell culture preparations, for each different experimental condition.

### 2.11. Statistical Analyses

Statistical analyses were performed using GraphPad Prism software version 9.3.0 for Windows (GraphPad Software, La Jolla, CA, USA). A non-parametric Mann–Whitney U-test was performed and differences with a *p*-value less than 0.05 were considered statistically significant (*: *p* < 0.05, **: *p* < 0.01, ***: *p* < 0.001).

## 3. Results

### 3.1. Modification of the HMEC-1 Cell Viability Following Individual and Combined Plasticizer Exposures

**Figure 2:**
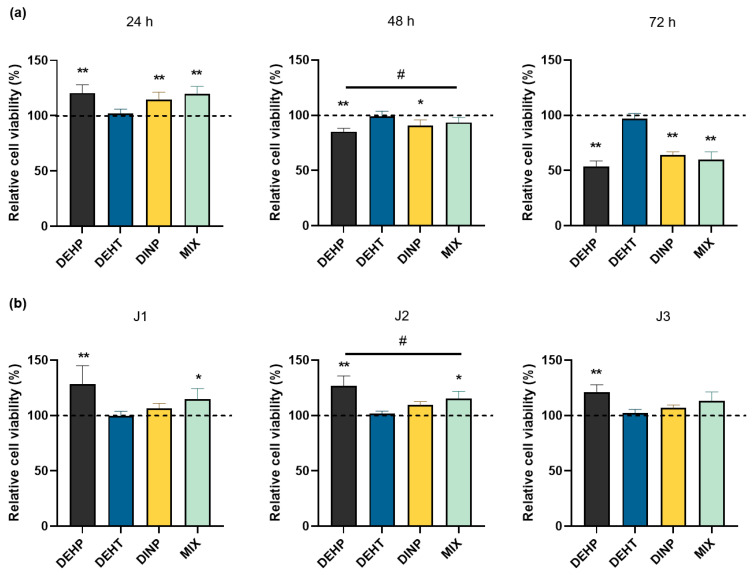
Comparison of cell viability in HMEC-1 cells following exposures to DEHP, DEHT and DINP (individual and combined): (**a**) for continuous exposures, HMEC-1 cells were incubated from 24 h to 72 h with DEHP, DEHT, DINP and the mixture (MIX); (**b**) for repeated exposures, HMEC-1 cells were incubated three hours per day between one and three days (J1, J2 and J3) with DEHP, DEHT, DINP and the mixture (MIX). Cell viability was measured by using MTT assay. For their comparison, results were normalized to the amount of proteins of the corresponding sample and were standardized with the solvent control. Results are expressed as the mean % ± SD (n ≥ 5), * *p* < 0.05 vs. DMSO, ** *p* < 0.01 vs. DMSO, # *p* < 0.05 vs. MIX.

Cell viability was evaluated in human microvascular endothelial cells (HMEC-1) using an MTT-spectrophotometric assay following exposure to DEHP, DEHT and DINP individually and combined (MIX), either continuously (Figure 2a) or repeatedly (Figure 2b).

Continuous exposure was conducted on confluent endothelial cells from 24 h to 72 h, at a concentration of 15 mg/L for each plasticizer for both individual and combined exposures. In Figure 2a, the results show significant modifications of the cell viability in continuously exposed cells compared with the control group. First, an increase was observed at 24 h of exposure for DEHP (121 ± 7%), DINP (115 ± 7%) and the mixture (120 ± 7%). It was followed by a decreasing cell viability, which was observed in a time-dependent manner for all the treatment groups, starting from 48 h of exposure for DEHP (85 ± 3%) and DINP (91 ± 5%), and from 72 h of exposure for the mixture (60 ± 7%). Cell viability was less affected by continuous exposure to DEHT.

For repeated exposure (Figure 2b), cells were treated 3 h per day during one (J1), two (J2) and three days (J3). Cells were exposed to a concentration of 15 mg/L for each plasticizer, for both individual and combined exposures. Between each treatment, plasticizer solutions were removed and replaced with fresh culture medium. For each repeated application, no significant changes in the cell viability were noticed in cells exposed either to DEHT or DINP compared to the control group. On the contrary, DEHP and the mixture displayed significant changes in comparison to the control group. Indeed, an increasing cell viability was reported with DEHP (mean values between 121 ± 7% and 128 ± 17%) like with the mixture (mean values of 115 ± 10%, except for J3 with a mean value of 107 ± 16% at the limit of the significance level).

### 3.2. Effects of Individual and Combined Plasticizer Exposures on HMEC-1 Cell Counting

**Figure 3:**
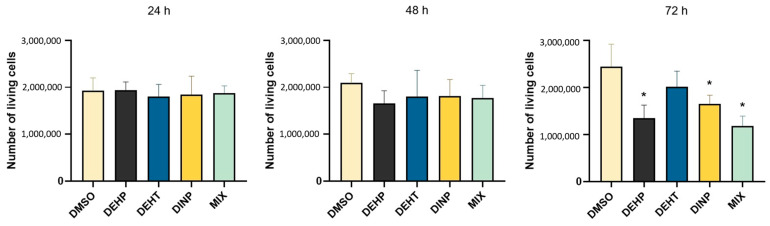
Comparison of the number of living HMEC-1 cells following exposures to DEHP, DEHT and DINP (individual and combined). HMEC-1 cells were incubated from 24 h to 72 h with DEHP, DEHT, DINP and the mixture. Results are expressed as the mean % ± SD (n = 4); * *p* < 0.05 vs. DMSO.

Cell counting is used to determine the number of living cells. Cell counting was performed using the trypan blue exclusion method following exposures to DEHP, DEHT, DINP and MIX during 24 h, 48 h and 72 h (Figure 3).

In Figure 3, the results show that the cell viability was not impaired following both 24 h and 48 h of exposure to the plasticizers for all the treatment groups compared to the solvent control. Cell viability differences were observed starting 72 h of exposure. A reduced number of living cells was observed with exposure to DEHP, DINP and the mixture, compared to the control group. On the contrary, DEHT did not significantly impair the number of living cells, regardless of the time of exposure.

### 3.3. Effects of Individual and Combined Plasticizer Exposure on the Total Intracellular Glutathione in HMEC-1 Cells

**Figure 4:**
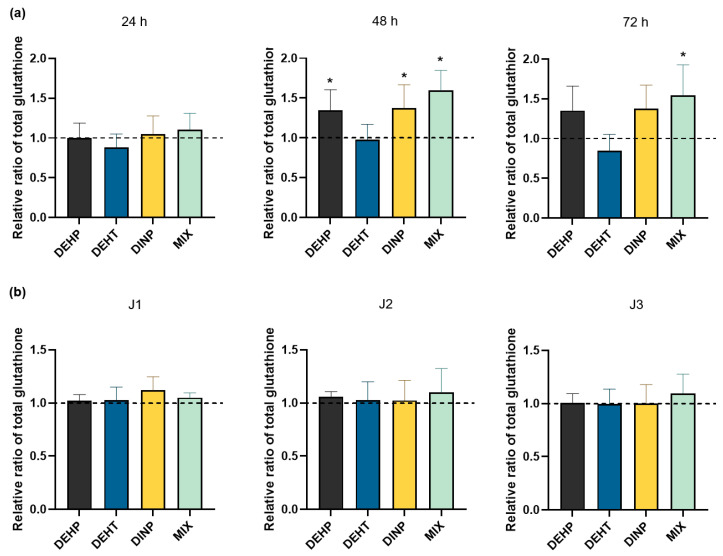
Comparison of total glutathione content in HMEC-1 cells following exposures to DEHP, DEHT and DINP (individual and combined): (**a**) for continuous exposures, HMEC-1 cells were incubated from 24 h to 72 h with DEHP, DEHT, DINP and the mixture; (**b**) for repeated exposures, HMEC-1 cells were incubated three hours per day between one and three days (J1, J2 and J3) with DEHP, DEHT, DINP and the mixture. For their comparison, results were normalized to the amount of proteins of the corresponding sample and were standardized with the solvent control. Results are expressed as the mean % ± SD (n ≥ 6), * *p* < 0.05 vs. DMSO.

Glutathione has a protective function against reactive species. The level of total glutathione is a marker of cellular oxidative stress due to its antioxidant capacity. The content of total glutathione was quantified in HMEC-1 cellular extracts with a spectrophotometric assay based on the reaction between GSH and the DTNB reagent following exposure to DEHP, DEHT and DINP individually and combined, either continuously (Figure 4a) or repeatedly (Figure 4b).

DEHT was the only plasticizer that did not show modifications in the content of total glutathione in cells for all treatment times (versus control group), as shown in Figure 4a. For the other treatment groups, no significant differences were observed at the first tested time of 24 h. The rise in antioxidant capacity in HMEC-1 cells was induced later following 48 h of exposure to DEHP, DINP and the mixture in comparison with the control group. The increasing content of total glutathione in cells was still observed following 72 h of exposure to the mixture (mean ratio of 1.54 ± 0.38) compared with the control group but it was reversed for cells exposed individually to both DEHP and DINP. Moreover, the effects of DEHP, DINP and the mixture on cells were all distinctives from DEHT at both 48 h and 72 h of exposure.

Considering the results of the content of total glutathione in cells repeatedly exposed to plasticizer(s), as shown in Figure 4b, no modifications were observed with any of the tested treatment groups, both individually and in mixture. It shows that the number of treatment repetitions had no effect on the content of total glutathione in cells exposed to the studied plasticizers regardless of the condition considered.

### 3.4. Effects of Individual and Combined Plasticizer Exposure on the Amount of Intracellular Adenosines in HMEC-1 Cells

**Figure 5:**
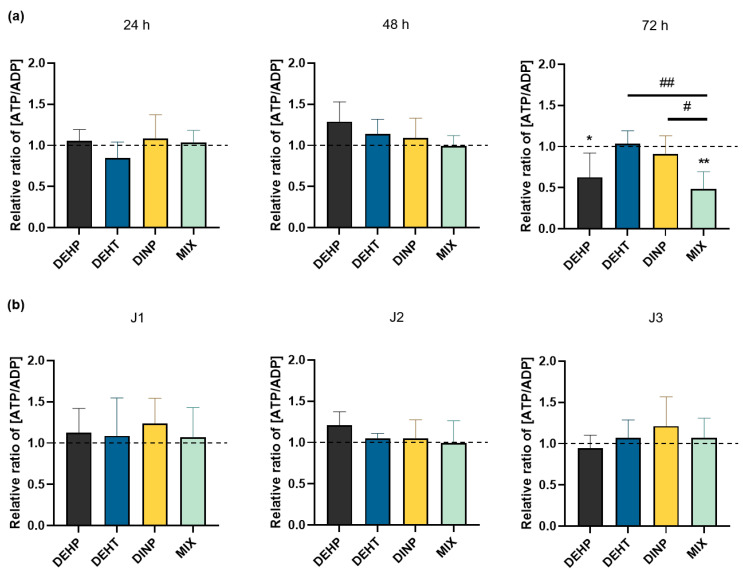
Comparison of the relative ratio of ATP/ADP in HMEC-1 cells following exposure to DEHP, DEHT and DINP (individual and combined): (**a**) for continuous exposures, HMEC-1 cells were incubated from 24 h to 72 h with DEHP, DEHT, DINP and the mixture; (**b**) for repeated exposures, HMEC-1 cells were incubated three hours per day between one and three days (J1, J2 and J3) with DEHP, DEHT, DINP and the mixture. For their comparisons, the relative ratio was standardized with the one of the solvent control for each sample of all treatment groups. Results are expressed as the mean % ± SD (n ≥ 4); * *p* < 0.05 vs. DMSO, ** *p* < 0.01 vs. DMSO, # *p* < 0.05 vs. MIX, ## *p* < 0.01 vs. MIX.

ATP is an important source of cellular energy. ATP is the core of physiological functions because it generates energy to cells through the cleavage of its phosphodiester bounds in ADP and AMP. The amounts of cellular adenosines were measured on HMEC-1 cellular extracts with HPLC-DAD following exposures to DEHP, DEHT and DINP individually and combined, either continuously (Figure 5a) or repeatedly (Figure 5b).

AMP was the only cellular adenosine that was not detected in the solvent control nor in the treatment groups, for both continuous and repeated exposure procedures (Figure 5a,b). The results for the cellular adenosines are presented as the ratio of ATP/ADP of the treatment group standardized with the ratio of the solvent control at the same exposure time. For the results of continuously exposed cells to plasticizers, as shown in Figure 5a, no effect was observed on the ratio of cellular adenosines at both 24 h and 48 h of exposure for all treatment groups. At 72 h of exposure, the cellular adenosines amounts were not modified in cells exposed to DINP and DEHT individually compared to the control group, contrary to the mixture. Indeed, the relative ratio of cellular adenosines dropped by half at 72 h. Similarly, cells exposed individually to DEHP showed a significant difference in comparison with the control group; its relative ratio of cellular adenosines lowered (0.63 ± 0.29) after 72 h of exposure compared to control DMSO.

Considering cells repeatedly exposed to the plasticizer(s) individually or in mixture, no modification of the relative ratio of ATP/ADP were observed with any of the tested treatment groups, as depicted in Figure 5b. It showed that the number of treatment repetitions had no effect on the adenosine amounts in cells exposed to the studied plasticizers.

### 3.5. Measurements of Mitochondrial Function in HMEC-1 Cells

**Figure 6:**
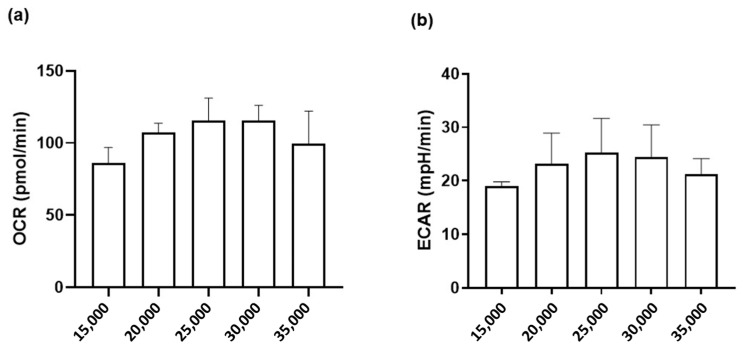
Measurement of oxygen consumption rate (OCR) and extracellular acidification rate (ECAR) as a function of cell seeding number. HMEC-1 were seeded into Seahorse plates 96 h before the measurements of (**a**) basal OCR and (**b**) ECAR. Data are the mean ± SD; n = 3–4 independent measurements.

To obtain a measurable OCR (a key parameter of oxidative phosphorylation, OXPHOS) and ECAR (an indicator of glycolysis) after plasticizer exposure, we first determined the optimal number of cells, as shown in Figure 6. OCR increased with a cell number between 15,000 and 25,000 and remained roughly stable at densities of 25,000 to 30,000 cells.

**Figure 7:**
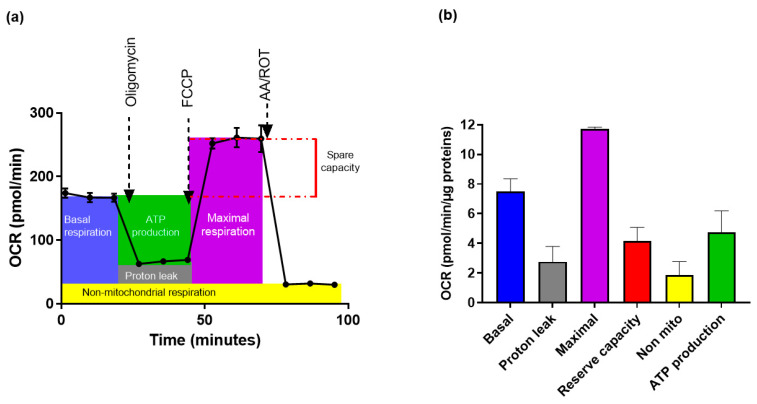
Oxygen consumption rate (OCR) for 25,000 cells seeded 96 h before the measurement of OCR and injection of inhibitors: (**a**) a representative diagram of the time course for measurement of OCR in HMEC-1 cells and the functional significance of the area under the curve; (**b**) the contribution of each of these parameters were calculated after normalization to protein amounts and the means ± SD from three independent measurements are plotted.

For subsequent experiments, the seeding of 25,000 cells per well was selected. OCR for 25,000 cells was then measured under the basal condition followed by the sequential addition of oligomycin, FCCP and the combination of AA and ROT, as described in the Materials and Methods. The AA and ROT-independent OCR allows to estimate the non-mitochondrial respiration and is subtracted from the other values (Figure 7a). Oligomycin inhibits ATP synthase and allows the measurement of OCR linked to ATP synthesis. In these cells, ATP-linked oxygen consumption represents about 70% of the mitochondrial respiration. The maximal OCR was obtained after the addition of the ionophore FCCP to uncouple mitochondria and allow calculating the spare respiratory capacity, after subtracting the basal OCR. This parameter represents an assessment of the supplemental ATP that can be generated by oxidative phosphorylation under stressful conditions. These measurements indicate that the cells are normally functioning in these experimental conditions (Figure 7b).

### 3.6. Effects of Plasticizers on Mitochondrial Function

Importantly, since 72 h induced cell mortality (except for the DEHT condition) and cell detachment during the runs, we performed mitochondrial evaluations after 24 h and 48 h of exposure. HMEC-1 cells were exposed 24 h or 48 h with the plasticizers before injection of oligomycin, FCCP and AA/ROT, as described above. After 24 h, only the DEHP-treated cells exhibited a change in energy metabolism, with a drastic decrease in basal OCR, a decreased maximal respiration and declined reserve capacity, and reduced ATP production (Table 1).

These observations were similar in mix-treated cells. After 48 h, these changes were exacerbated following DEHP exposure. Decreased basal OCR, reserve capacity and ATP production were also observed after DINP exposure, although the decreasing magnitudes were smaller than those observed with DEHP. We also observed a decrease in non-mitochondrial OCR after 48 h of exposure to DINP. The combination of plasticizers did not amplify the observed response with DEHP alone.

**Figure 8:**
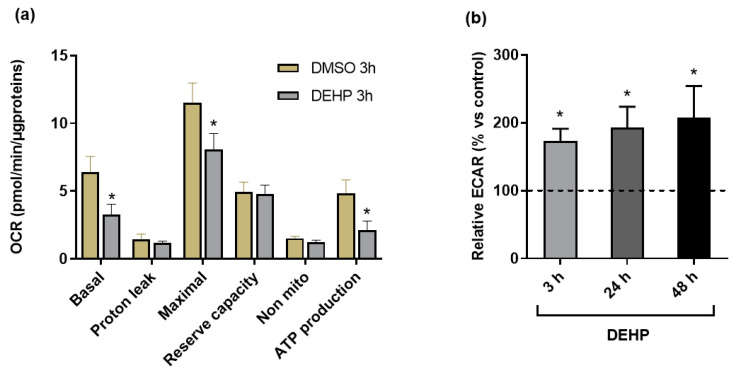
Effect of DEHP on OCR and ECAR: (**a**) OCR measurements after 3 h of exposure to DEHP compared to the vehicle controls; (**b**) ECAR measurement after 3 h, 24 h or 48 h of exposure to DEHP. * *p* < 0.05 vs. vehicle controls.

The alteration in energy metabolism observed after 24 h and 48 h of DEHP exposure was observed as early as 3 h of incubation (Figure 8a) and was associated with an increase in acidification, whatever the time of exposure (Figure 8b).

## 4. Discussion

Exposure to DEHP has been documented to be a risk factor for cardiovascular diseases [25,26,49]. Although endothelial cells are directly exposed to circulating plasticizers, few studies have evaluated the endothelial toxicity of plasticizers, and to a lesser extent, mixture effects [28,30,32,41,50]. Considering that DEHP regulation paved the way to its substitution by other plasticizers, such as DINP and DEHT, it is necessary to evaluate their effects on the vascular system owing to the lack of data. Therefore, the aim of this study was to evaluate the in vitro endothelial toxicity of DEHP, DINP and DEHT, both individually and all mixed together. Each plasticizer was at a concentration of 15 mg/L in all the solution tested, which corresponds to about 40 µM per plasticizer. These three plasticizers were selected based upon previous experiments carried out in our laboratory from gloves [6]. We performed intermittent episodes of 3 h of exposure separated by recovery periods to investigate the recovery capacity of endothelial cells after phthalate exposure. Phthalates are ubiquitous molecules and human exposure can occur through multiple pathways of exposure, so although these molecules have a short half time, humans can be continually exposed, so we have also carried out continuous exposure. The studied plasticizers were tested on cells following these two distinctive exposure procedures to evaluate respectively continuous (24, 48 and 72 h) and repeated exposures (J1, J2 and J3) for the following toxicological endpoints: cell viability, total intracellular glutathione (total glutathione) content and cellular adenosine amounts.

In this study, the more pronounced effects of plasticizers were observed with continuous exposure, as shown in the cell viability measurements. Unexpectedly, an increase in cell viability for DEHP, DINP and the mixture of plasticizers was observed following 24 h of exposure, which preceded a subsequent decrease in a time-dependent manner. In the literature, comparable conditions of time and concentration resulted in a diminishing cell viability, as reported for vascular endothelial cells exposed to MEHP, the main metabolite of DEHP, in HUVEC (from 12.5 µM) and in EA.hy926 (from 100 µM MEHP) [28,30,32]. On the other hand, it is of interest to note that an increasing cell viability trend was reported on a non-endothelial cellular model for DEHP (250 µM) and DINP (62.5 µM), tested separately on a hepatocarcinoma cell line at an exposure time of 24 h [51]. Overall, the time-dependent decrease observed in our study for DEHP and DINP was in line with this study, with more marked effects at 72 h comparatively to 48 h of exposure [51]. Cell viability could be explained by cell proliferation as its decrease was reported for DEHP on human corneal endothelial cells, with tested concentrations higher than those in the current study (from 100 µM) [52]. Based on our results, it is not possible to determine whether viability alterations are a consequence of cell division capacity. However, an MTT assay does not necessarily reflect cell viability; it could also be related to a metabolic and mitochondrial reprogramming induced by a stress, as it has been described to contribute to MTT cellular reduction [53]. Among the two DEHP substitutes, only DEHT showed no alteration on cell viability, which is in agreement with a study conducted on a fibroblast cell line testing higher concentrations (0.01–0.1 mg/mL) [54]. The results obtained with repeated exposure surprisingly did not worsen the cell viability as expected. Quite comparable results were obtained, regardless of the number of repeated exposures. One plausible hypothesis is that intermittent incubation of cells with the plasticizers, and with the renewal of the culture medium, interrupts cell exposure between each treatment period and therefore may enable cells to recover.

By comparing cell counting and cell viability, the results did not show corresponding effects between these parameters. The increasing cell viability measured with the MTT assay following 24 h of exposure was not observed during cell counting. As the results are inconsistent, it supports the hypothesis that plasticizers induced a metabolic and mitochondrial reprogramming in HMEC-1 cells rather than a cellular proliferation. By taking this into consideration, it could be presumed that renewal of the culture medium does not result in cellular metabolic perturbations due to the absence of effects seen from the MTT assay with the repeated exposures.

It is of interest to note that cell viability indirectly reflects plasticizer-induced cytotoxicity, and so our results could be explained by cellular physiological perturbations, as a redox imbalance has been reported in plasticizer-induced cardiovascular pathogenesis [21]. Glutathione is an endogenous antioxidant that plays a role in cytoprotection. In the present study, for repeated exposures, there was no distinction between the tested conditions nor between the number of exposures on the total glutathione content. Impairments were noted in the present study with continuous exposure. While a transient increase in the total glutathione content occurred at 48 h of exposure for DEHP and DINP (both individually), a steady rise was observed up to 72 h of exposure when all three plasticizers are in mixture. It demonstrates that the plasticizers displayed different cytotoxicity when they were mixed by inducing modifications of the glutathione content. Altogether, these results reflect a cellular stress caused by plasticizers which could result from the production of reactive species. In this regard, it would be interesting to distinguish which glutathione form contributes to the elevation of the total glutathione content; so, determining the ratio of the reduced form (GSH) to oxidized form (GSSG) can be a useful indicator. Previous experimental studies described, for instance, GSH depletion in HUVEC along with increased ROS production following 24 h of exposure to MEHP (about 20 µM) [30,55].

The implications of ROS in vascular diseases have been previously stated and the results underlined that endothelial mitochondria are a target of the deleterious effects of ROS. The loss of the potential mitochondrial membrane was depicted by several in vitro studies, which showed plasticizer-induced ROS production in endothelial cells [28,30,32]. Despite this, data on the effects of plasticizers on mitochondrial respiration are scarce. That is why mitochondrial respiration was assessed in the current study to further understand how plasticizers could impair this function, using a Seahorse metabolic flux assay to evaluate oxygen consumption rates and extracellular acidification rates. It was decided to focus on continuously exposed endothelial cells owing to the noticeable cytotoxicity compared to the repeated exposure. As far as we know, we demonstrate for the first time that DEHP or DINP induced an OXPHOS defect on vascular endothelial cells. The absence of mitochondrial dysfunction after DEHT exposure supports its non-cytotoxicity to endothelial cells. The mixture and DEHP present comparable effects on mitochondrial respiration, which led us to think it is the main contributor to the mixture effects, or at least more so than DINP. Overall, plasticizer exposure represents a stressful condition for mitochondria, with the disruption of both basal and reserve capacity. As seen with the decrease in mitochondrial ATP production, mitochondria seem not to be able to face energy demand when exposed to DEHP, DINP and the mixture. The mitochondrial respiration decreased as early as 3 h for the DEHP and lowered between 24 h and 48 h respectively for the mixture and for DINP. Beyond 48 h, these effects were not assessed due to the observation of cell detachment at 72 h of exposure, which is in line with the drop of the cell viability previously described. The early mitochondrial ATP depletion for the DEHP and the mixture was not consistent with the effects observed for the adenosines levels, which declined on a later stage at 72 h of exposure. These results strengthen the hypothesis of a plasticizer-induced metabolic reprogramming because mitochondrial ATP production and the ATP/ADP ratio declined in a time-delayed manner. This hypothesis is strengthened by the increase in acidification rate, which reflects a shift towards an anaerobic glycolytic metabolism.

Impairment of the oxidative phosphorylation was previously described on skeletal muscle cells exposed to MEHP; the authors reported a degradation of complex I, which was triggered by ROS production [56]. In the present study, the redox imbalance was found subsequently to the mitochondrial respiration alteration, but it is not excluded that early mitochondrial ROS production occurs. The short-term effect of DEHP on mitochondrial respiration has not yet been evaluated to our knowledge. In the present study, results of short-term exposure provide additional information. Indeed, the sudden decrease in mitochondrial synthesis of ATP occurred as early as 3 h of exposure with DEHP. Interestingly, it did not directly affect cellular physiological functions when comparing the first 3 h of the repeated exposure, as no modifications were noted. Considering these kinetic effects, it reinforces the idea that endothelial cells are not only capable to adapt to cellular stress induced by plasticizers but also are quickly responsive. It is likely to believe that this adaptation goes through metabolic pathways, leading to rapid changes to effectively maintain the cellular processes. By gathering data from the literature on endothelial cell metabolism in vascular diseases (angiogenesis and atherosclerosis), the studies revealed adaptable cell metabolism [57,58]. Physiologically, glycolysis and mitochondrial respiration are necessary for HUVEC proliferation [59]. However, a metabolic shift in endothelial cells in favor of fatty acid oxidation and/or glycolysis occurred during cardiovascular pathogenesis in response to endogenous signals, oxidized LDL and angiogenic factors [57,58].

By analogy, an endothelial metabolic shift caused by circulating plasticizers in blood could be presumed because the dysfunctional mitochondrial respiration was combined with an increased acidification, supporting a possible shift to anaerobic metabolism, resulting in lactate production. In the metabolic shift observed during angiogenesis and atherosclerosis, the implications of PPAR and oxidative stress were proposed [58,60]. Considering that plasticizers are known as PPAR ligands as well as oxidative stress inductors, their implication in the metabolic shift might be plausible in endothelial cytotoxicity [30,47,61]. However, based on our results, it is not possible to determine the cause of the mitochondrial dysfunction because it was not investigated. Hence, further experiments are needed to confront these hypotheses.

## 5. Conclusions

In summary, the present study showed that plasticizers induced an endothelial dysfunction by targeting the mitochondrial respiration with a depletion of ATP production, which occurred earlier for DEHP compared to DINP and the mixture. On the contrary, DEHT did not show any cytotoxicity. According to the delayed effects between the cellular and the mitochondrial parameters, these results suggest that a mechanism of adaptation takes place in endothelial cells to face plasticizer-induced cellular stress, leading to metabolic reprogramming to effectively maintain their cellular processes. To conclude, this study provides new insights into the adverse effects of plasticizers on endothelial cells and underlines the importance of mitochondrial evaluation in the safety screening of plasticizers.

## Figures and Tables

**Table 1:** Effects of plasticizers on mitochondrial function.

		Control	DEHP	DEHT	DINP	Mix
Basal	24 h	8.4 ± 0.9	4.8 ± 0.5 *	9.8 ± 1.0	6.8 ± 1.1	5.4 ± 0.6 *
	48 h	7.5 ± 1.6	3.5 ± 0.7 **^#^	8.8 ± 2.6	5.2 ± 0.6 **^$^	3.8 ± 1.2 *
Proton leak.	24 h	2.3 ± 0.5	1.9 ± 0.2	2.5 ± 0.3	1.9 ± 0.4	1.9 ± 0.4
	48 h	2.0 ± 0.5	1.7 ± 0.4	2.3 ± 0.7	1.7 ± 0.3	1.4 ± 0.4
Maximal	24 h	13.3 ± 1.9	6.8 ± 0.9 *	15.2 ± 1.1	10.6 ± 1.7	7.8 ± 0.7 *
	48 h	12.6 ± 3.2	4.8 ± 0.7 **^#^	12.4 ± 1.6	7.8 ± 0.8 **	5.1 ± 1.5 *
Reserve capacity	24 h	5.0 ± 1.1	2.1 ± 0.7 *	5.4 ± 0.7	3.8 ± 1.2	2.4 ± 0.4 *
	48 h	5.1 ± 1.6	1.3 ± 0.2 **	5.6 ± 2.2	2.6 ± 0.6 **^$$^	1.3 ± 0.3 *
Non-mito.	24 h	2.0 ± 0.5	1.4 ± 0.6	2.0 ± 1.0	1.6 ± 0.8	1.6 ± 0.8
	48 h	1.6 ± 0.4	1.4 ± 1.1	1.6 ± 0.4	0.9 ± 0.2 **	0.9 ± 0.2 *
ATP prod.	24 h	6.1 ± 0.8	2.6 ± 0.9 *	7.3 ± 1.0	4.9 ± 1.1	3.5 ± 0.8 *
	48 h	5.5 ± 1.2	1.8 ± 0.6 **	6.6 ± 2.0	3.5 ± 0.6 **^$^	2.4 ± 0.8 *

Bis-(2-ethylhexyl) phthalate (DEHP), bis-(2-ethylhexyl) terephthalate (DEHT) and diisononyl phthalate (DINP). The values represent the means ± SD of four independent measurements. * *p* < 0.05 vs. DMSO; ** *p* < 0.01 vs. DMSO; ^#^ *p* < 0.05 24 h vs. 48 h; ^$^ *p* < 0.05 DEHP vs. DINP; ^$$^ *p* < 0.01 DEHP vs. DINP.

## Data Availability

Not applicable.
